# Vertical transmission potential of SARS-CoV-2 from infected mother to twin neonates

**DOI:** 10.2217/fvl-2020-0324

**Published:** 2021-06-22

**Authors:** Liangyu Peng, Suliman Khan, Ashaq Ali, Saeed Ahmed, Liaqat Ali, Guang Han, Yang Jing

**Affiliations:** 1 ^1^Department of Gynecology & Obstetrics, Renmin Hospital of Wuhan University, Wuhan, 430010, China; 2 ^2^The Second Affiliated Hospital of Zhengzhou University, Zhengzhou, China; 3 ^3^State Key Laboratory of Virology, Wuhan Institute of Virology, Chinese Academy of Sciences, Wuhan, 430070, China; 4 ^4^Department of Biological Sciences, National University of Medical Sciences, Rawalpindi, 46000, Pakistan; 5 ^5^University of Chinese Academy of Sciences, Beijing, 100049, China; 6 ^6^Department of Radiation Oncology, Hubei Cancer Hospital, Tongji Medical College, Huazhong University of Science & Technology, Wuhan, 430070, China; 7 ^7^Reproductive Medicine Center, Renmin Hospital of Wuhan University, Jiefang Road 238, Wuhan, 430060, China.

**Keywords:** COVID-19 infection, pregnancy outcomes, twins, vertical transmission

## Abstract

**Background:** Limited details are available regarding the vertical transmission potential of COVID-19 infection in pregnant women. The authors' current study aimed to report the vertical transmission potential of COVID-19 infection in a woman pregnant with twins. **Case description:** The authors report the case of a 27-year-old woman infected with SARS-CoV-2. The patient was pregnant with dichorionic diamniotic fraternal twins and admitted to Renmin Hospital of Wuhan University, Wuhan, China. After undergoing a cesarean section, the patient gave birth to premature twins, who tested positive for COVID-19 infection. **Interpretation:** Findings from this case suggest a possibility of intrauterine infection caused by vertical transmission in a woman infected with COVID-19.

Pregnancy elicits various anatomical and physiological changes in women that make them vulnerable to respiratory diseases [1]. It is well known that maternal physiological adjustments to pregnancy increase the risk of developing severe conditions in response to viral infections such as influenza. Preliminary data indicate that the prognosis of SARS-CoV-2 infection in pregnant women may also be more severe [2].

As the number of SARS-CoV-2 upsurges worldwide, numerous studies emphasized the need of proper investigations addressing SARS-CoV-2-positive women. Pregnant women with SARS-CoV-2 are more challenging to manage than women who are not pregnant; thus, transparent and comprehensive reporting of all cases of COVID-19 pregnancies is essential [3]. The potential for vertical transmission was also previously revealed by identifying SARS-CoV-2 RNA and the expression of N and S proteins in the placenta of pregnant COVID-19 patients [4]. To better prepare for the health consequences, studies related to the spread and vertical transmission of COVID-19 are essential. In this study, the authors present the case of a woman pregnant with twins who contracted COVID-19 of unknown etiology. The delivered premature newborns were diagnosed with COVID-19 soon after birth.

## Methods

The authors reviewed clinical records and laboratory findings for neonates and clinical records, laboratory findings and chest computed tomography (CT) scans for pregnant women. Samples were tested for SARS-CoV-2 with the kit recommended by the Chinese Center for Disease Control and Prevention (GeneoDx, Shanghai, China) following WHO guidelines for quantitative real-time PCR. The quantitative real-time PCR kits were purchased from HEALTH Gene Technologies Co., Ltd. (Ningbo, China). All samples (meconium, pharyngeal swab and sputum) were processed at the Department of Clinical Laboratory (Renmin Hospital, Wuhan University, Wuhan, China).

### Case description

A previously healthy 27-year-old woman pregnant with twins had routine prenatal care, screening and treatment at Renmin Hospital. At the gestational age of 30 weeks 6 days, the patient was diagnosed with premature rupture of membranes (PROM) in the elder fetus and admitted to Renmin Hospital of Wuhan University on 5 February 2020.

The patient received routine treatment for inhibition of uterine contractions, fetal lung maturation and infection prevention. To prevent umbilical cord prolapse, the patient underwent cesarean section at a gestational age of 31 weeks 2 days on 8 February 2020, and premature twin baby girls (neonate 1 and neonate 2) with lower than normal weight were delivered. Up to the performance of the cesarean section, the patient was found to have no history of epidemiological exposure to COVID-19 and had no symptoms related to COVID-19. Moreover, she did not have underlying diseases, including diabetes, chronic hypertension, cardiovascular disease, gestational hypertension or pre-eclampsia. However, after the cesarean section, the patient tested positive for COVID-19 infection, though she remained asymptomatic.

Neonate 1 was born with abnormal cardiopulmonary function as well as heart and respiratory rates. Apgar scores were 3 at 1 min and 4 at 5 min, weight was 1580 g and length was 46 cm (Table 1). Nucleic acid tests (real-time PCR) for pharyngeal swab and meconium tested positive for SARS-CoV-2. By contrast, neonate 2 responded well, with Apgar scores of 9 at 1 min and 10 at 5 min. The heart rate was normal, weight was 1580 g and length was 48 cm. However, pharyngeal swab, sputum and meconium tested positive for SARS-CoV-2. Both neonates were provided with pediatric care at Wuhan Children's Hospital. The doctors and staff were routinely checked for COVID-19, and all doctors and staff members in contact with the patient were found to be negative for SARS-COV-2. In the course of routine postdelivery investigation of the neonates, the nucleic acid test turned negative within 2–3 weeks after the first diagnosis, and breastfeeding was started soon after the babies and mother became negative for SARS-CoV-2 (three consecutive negative quantitative PCR tests). Subsequently, the babies were discharged from the hospital.

**Table 1.** Summary of clinical and laboratory characteristics of neonates infected with COVID-19.

Clinical details	Neonate 1	Neonate 2
Birth weight (kg)	1.58	1.58
Birth length (cm)	46	48
Apgar score (1–5 min)	3–4	8–9
Normal fetal heart rate	No	Yes
Normal ultrasound results	Yes	Yes
Preterm delivery	Yes	Yes
Infected with COVID-19	Yes	Yes
Neonatal pneumonia	No	No
Meconium qRT-PCR for COVID-19 infection	Positive	Positive
Pharyngeal swab qRT-PCR for COVID-19 infection	Positive	Positive
Sputum qRT-PCR for COVID-19 infection	Negative	Positive

qRT-PCR: Quantitative real-time PCR.

## Results & discussion

The authors report clinical data from an atypical case of COVID-19 infection in a pregnant woman who gave birth to infected twin baby girls. The clinical characteristics (Table 2) of this patient during pregnancy were similar to those of noninfected pregnant women. Recently, some reports [5] have stressed the importance of CT scan imaging and serum antibody response in the early detection of SARS-CoV-2 in asymptomatic pregnant patients. Similarly, as per Chinese Center for Disease Control and Prevention recommendations, all COVID-19 patients underwent CT scan and other necessary investigations, and the pregnant women did not develop any symptoms of viral pneumonia and CT scan images did not show significant abnormalities in the lungs. Such asymptomatic patients with COVID-19 infection have been reported previously [6]. The authors' results suggest that PROM could be the result of COVID-19 infection in pregnant women; however, the authors cannot rule out the possibility that PROM may be linked to other biological processes or intrauterine infections [7]. Previously, the risk of SARS-CoV-2 transmission from mother to baby and the significance of clinical investigations for such as cases are extensively highlighted [3,8]. Herein the detection of SARS-CoV-2 in first-pass meconium samples of the neonates indicated the possibility of intrauterine vertical transmission of infection.

**Table 2.** Summary of clinical and laboratory characteristics of pregnant woman infected with COVID-19.

Clinical characteristics	Pregnant woman
Date of hospital admission	5 February 2020
Age (years)	27
Gravidity and parity	G1P1
Gestational age on admission, weeks + days	30 + 6
Gestational age on delivery, weeks + days	31 + 2
Fetal stay in the uterus, onset of pneumonia until birth (days)	Unknown
Source of SARS-CoV-2 infection	Unknown
History of contact with family member or patient	No
Maximum body temperature	36.7
Complications	No
Signs and symptoms of COVID-19	No
Nucleic acid/antibody	Positive
Infected with COVID-19	Yes
Treatment/therapy	No

**Laboratory characteristics**	
White blood cell count (×10^9^ cells/l)	8.27
Lymphocyte count (×10^9^ cells/l)	1.28
C-reactive protein concentration (mg/l)	12.1
ALT, U/l	7
AST, U/l	15
qRT-PCR confirmation for COVID-19 infection	Positive

ALT: Alanine transaminase; AST: Aspartate aminotransferase; qRT-PCR: Quantitative real-time PCR.

Pregnant women are in an immunosuppressive state; therefore, they are thought to be susceptible to respiratory pathogens [9]. In the current study, laboratory tests confirmed infection in the patient; however, lymphocytes, leukocytes, ALT and AST were found to be within the normal range. An increase in C-reactive protein was observed, which might be an adverse clinical manifestation [10,11]. Although the pregnant woman was diagnosed with PROM, no risk factors, including pre-eclampsia, history of cesarean section or fetal distress, were observed. Previously, Chen *et al.* reported that one patient among nine pregnant women had PROM and suspected intrauterine infection [12]. Moreover, Khan *et al.* reported the possibility of adverse pregnancy outcomes in women infected with COVID-19 [13]. Similarly, the occurrence of PROM and detection of SARS-CoV-2 in meconium samples indicated that COVID-19 was linked to adverse pregnancy outcomes. The authors of the current study report abnormal heart function in neonate 1, which can be an outcome of COVID-19 infection.

Unfortunately, the authors could not test amniotic fluid and cord blood, which are important in studying the vertical transmission of infection. The authors' results suggested that the intrauterine fetal infections occurred as a result of COVID-19 infection at some point during the early stages of the third trimester. The authors' current study is limited by small sample size, unavailability of detailed laboratory testing for cord blood and amniotic fluid and unknown etiology of the COVID-19 infection. Therefore, further investigations are necessary to evaluate whether COVID-19 can vertically transmit and cause PROM. In summary, both babies and their mother tested positive for COVID-19 infection, suggesting the possibility of intrauterine vertical transmission of COVID-19 infection in women who deliver preterm twin babies.

**Executive summary**

- The vertical transmission potential of COVID-19 infection is of great importance.
- The authors report that twin neonates and their mother were found to be infected with SARS-CoV-2.
- Although the woman discussed in this case report was asymptomatic, one of the neonates was diagnosed with lung dysfunction.
- The investigations in this case report indicate that COVID-19 may cause lung problems, premature delivery and related symptoms.
- The findings from this case report suggest the possibility of intrauterine vertical transmission of COVID-19 infection in twin neonates.
